# Genomes of “*Spiribacter*”, a streamlined, successful halophilic bacterium

**DOI:** 10.1186/1471-2164-14-787

**Published:** 2013-11-13

**Authors:** Mario López-Pérez, Rohit Ghai, Maria Jose Leon, Ángel Rodríguez-Olmos, José Luis Copa-Patiño, Juan Soliveri, Cristina Sanchez-Porro, Antonio Ventosa, Francisco Rodriguez-Valera

**Affiliations:** 1 Evolutionary Genomics Group, División de Microbiología, Universidad Miguel Hernández, Apartado 18, San Juan 03550, Alicante, Spain; 2 Department of Microbiology and Parasitology, Faculty of Pharmacy, University of Sevilla, Sevilla 41012, Spain; 3 Department of Biomedicine and Biotechnology, Faculty of Pharmacy, University of Alcalá, Alcalá de Henares 28871 Madrid, Spain

**Keywords:** Halophilic bacteria, Xanthorhodopsin, Hypersaline, Saltern, *Spiribacter*, Moderate halophile

## Abstract

**Background:**

Thalassosaline waters produced by the concentration of seawater are widespread and common extreme aquatic habitats. Their salinity varies from that of sea water (*ca*. 3.5%) to saturation for NaCl (*ca*. 37%). Obviously the microbiota varies dramatically throughout this range. Recent metagenomic analysis of intermediate salinity waters (19%) indicated the presence of an abundant and yet undescribed gamma-proteobacterium. Two strains belonging to this group have been isolated from saltern ponds of intermediate salinity in two Spanish salterns and were named “*Spiribacter*”.

**Results:**

The genomes of two isolates of “*Spiribacter*” have been fully sequenced and assembled. The analysis of metagenomic datasets indicates that microbes of this genus are widespread worldwide in medium salinity habitats representing the first ecologically defined moderate halophile. The genomes indicate that the two isolates belong to different species within the same genus. Both genomes are streamlined with high coding densities, have few regulatory mechanisms and no motility or chemotactic behavior. Metabolically they are heterotrophs with a subgroup II xanthorhodopsin as an additional energy source when light is available.

**Conclusions:**

This is the first bacterium that has been proven by culture independent approaches to be prevalent in hypersaline habitats of intermediate salinity (half a way between the sea and NaCl saturation). Predictions from the proteome and analysis of transporter genes, together with a complete ectoine biosynthesis gene cluster are consistent with these microbes having the salt-out-organic-compatible solutes type of osmoregulation. All these features are also consistent with a well-adapted fully planktonic microbe while other halophiles with more complex genomes such as *Salinibacter ruber* might have particle associated microniches.

## Background

The development of techniques to directly study genes in the environment, first by PCR and cloning of 16S rRNA genes and later by metagenomics has provided a more realistic view of the microbial community structure of natural environments. Historically, a gap between cultured microbes and the real natural diversity was recognized and expressed as the “great plate anomaly” [1]. We are now in a better position to assess the selective bias introduced by nutrient-rich laboratory media. “Classical” marine bacteria, such as *Vibrio* species, easily isolated in pure culture, are now recognized as rare inhabitants selected by laboratory conditions [2]. On the other hand, the most abundant marine microbes such as *Candidatus* Pelagibacter, are extremely difficult to retrieve in pure culture and are still largely studied by direct sequencing of the biomass or, at best, of a few isolated strains [3]. Rather than such unexpected findings remaining restricted to the marine habitat, they rapidly turned out to be the norm, especially in the case of aquatic systems, e.g. the high abundances of low GC Actinobacteria in freshwater [4] and even in the ocean [5] and many other groups that are just starting to be known and studied [6,7]. A similar discovery was made regarding hypersaline environments. The classical extreme halophiles that have been studied for decades such as *Halobacterium* or *Haloarcula* were barely detectable by molecular approaches in saturated brines [8,9]. Instead, slow growing and extremely fastidious to isolate microbes, such as *Haloquadratum walsbyi*, have shown to be dominant worldwide in neutral saturated brines [10,11]. Recently, a new group of extremely small archaea (the Nanohaloarchaea) were discovered by metagenomic approaches to be also relatively abundant in saturated brines, but no culture of these microbes is yet available [12-15].

In a large metagenomic study carried out in a solar saltern in which different salinities were studied by direct 454 pyrosequencing [12], it came as no surprise when in intermediate salinities (19%, about half a way between seawater salinity, 3.5% and saturation, 37%) the classical moderately halophilic bacteria such as *Salinivibrio* or *Halomonas*[16] turned out to be nearly absent. The few studies carried out by PCR and cloning of 16S rRNA [9] had already indicated the presence of bacteria distantly related to *Nitrococcus mobilis*. Metagenomic assembly provided many contigs that clearly belonged to gamma-proteobacterial cells that gave consistent hits to the genomes of both *Nitrococcus mobilis* Nb-231 and *Alkalilimnicola ehrlichii* MLHE-1. Assembly of the metagenome allowed to reconstitute large genomic fragments and indicated that this was a new microbe (or group of microbes) distantly related of the previously mentioned.

In an attempt to retrieve this new microbe, oligotrophic media were designed to isolate bacteria from a *ca*. 20% salinity saltern ponds in Isla Cristina, Huelva (Spain) and from the large number of colonies screened, a microbe with 95% identity of the 16S rRNA gene to *Alkalilimnicola* could be isolated. Independently, a sample from the intermediate salinity saltern in Santa Pola also gave a similar isolate. Both microbes, like most environmentally successful aquatic microbes, are extremely slow growing and fastidious to maintain in the laboratory. Their genomes have now been sequenced and assembled and they have proven to be representatives of the bacteria that were dominant in the 19% saltern pond metagenome. Actually they seem to be very abundant at all medium salinity (15-25%) neutral hypersaline waters for which metagenomes are available.

## Results and Discussion

Two strains, M19-40 and UAH-SP71, were isolated from saltern ponds of intermediate salinity (see methods) in South west and South east Spain (Isla Cristina and Santa Pola) respectively. Both microbes are representatives of a novel group of *Ectothiorhodospiraceae* that by metagenomics appeared to be the dominant bacterial group in saltern ponds of intermediate salinity [12]. By 16S rRNA sequencing they were tentatively assigned to a new genus “*Spiribacter*” and two separate species “*Spiribacter salinus*” M19-40 and “*Spiribacter*” sp. UAH-SP71. A complete taxonomic description is being prepared (Leon et al., in preparation). The genomes of the two isolates have now been fully sequenced and assembled into a single contig (Table 1).

**Table 1:** **General features of the two new genomes compared with *A. ehrlichii***

	**“ *Spiribacter salinus *” M19-40**	**“ *Spiribacter *” sp. UAH-SP71**	***A*. *ehrlichii *** **MLHE-1**
**Size (bp)**	1,739,487	1,926,631	3,275,944
**GC content (%)**	62,7	63,9	67,5
**Contigs**	1	1	1
**Protein coding genes**	1706	1874	2865
**rRNA operons**	1	1	2
**tRNAS**	45	44	48
**Hypothetical proteins**	173	199	637
**Functions assigned**	1533	1675	2228
**IS elements**	7	5	20
ANI (%)^*^	--	77,3	68,0

^*^ANI, average nucleotide identity [21].

### General characteristics and comparison with other *Ectothiorhodospiraceae*

**Figure 1:**
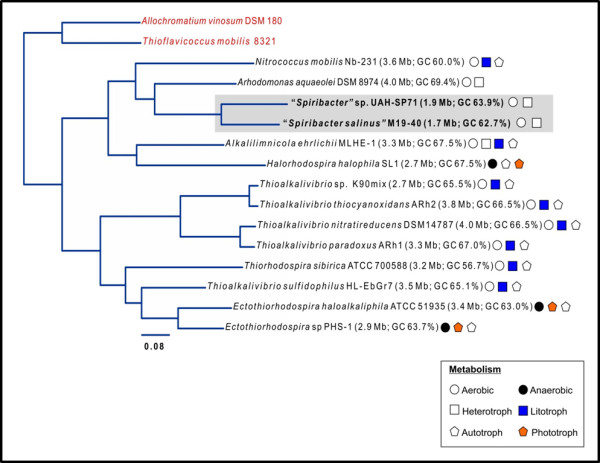
**Phylogenetic tree constructed using a concatenate of 277 conserved proteins in all genomes of available *Ectothiorhodospiraceae *genomes. ***Allochromatium vinosum* DSM 180 and *Thioflavicoccus mobilis* strain 8320, belonging to *Chromatiaceae* were used as outgroups and are shown in red. Metabolic characteristics are indicated next to the names and a key is provided at the bottom right. “*Spiribacter*” genomes are highlighted in a grey box.

**Figure 2:**
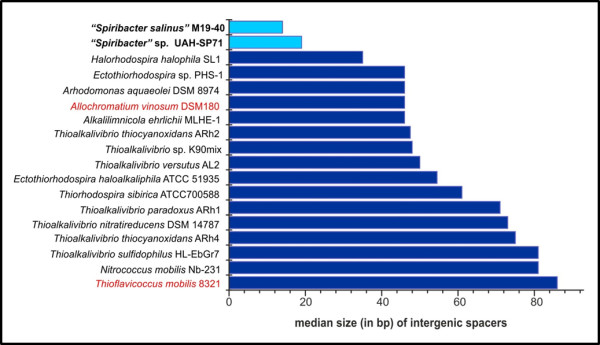
**Comparison of intergenic spacer size in the *Ectothiorhodospiraceae *available genomes.** Two genomes from *Chromatiaceae* are highlighted in red.

**Figure 3:**
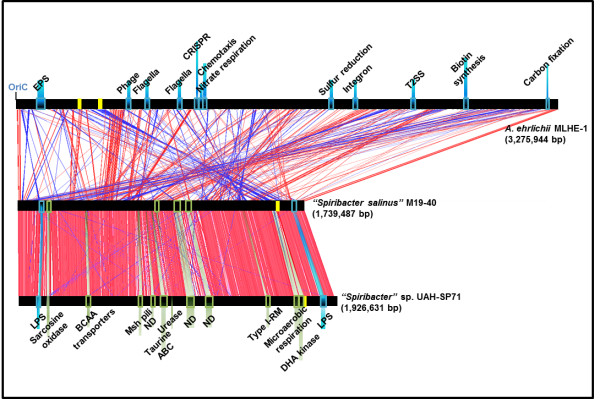
**Alignment of the “*Spiribacter*” genomes and the closest available genome of *Alkalilimnicola ehrlichii *MLHE-1.** Each genome is represented linearized starting from the OriC. Some genomic regions relevant to lifestyle present in *A. ehrlichii* MLHE-1 and completely missing in “*Spiribacter*” are highlighted as blue rectangles. Genomic islands of very different genetic make up between both “*Spiribacter*” genomes are indicated below, in blue when present in both (but containing different genes) and green when present in one of the strains only. Locations of rRNA genes of each genome are indicated in yellow.

The phylogenomic relationships of “*Spiribacter*” with other members of the family *Ectothiorhodospiraceae* that have been completely sequenced are shown in Figure 1. *Allochromatium vinosum* DSM 180 and *Thioflavicoccus mobilis* 8321, two purple sulfur marine bacteria which belong to the family *Chromatiaceae*, were used as outgroup. The family *Ectothiorhodospiraceae* is composed of purple sulfur photosynthetic bacteria that are in most cases halophilic and also often alkaliphilic [17]. Many of the most classical isolates come from alkaline hypersaline lakes [18]. The results confirm the initial phylogeny described by 16S rRNA gene sequence comparison (Additional file 1: Figure S1). Both strains appear monophyletic and only distantly related to the closest neighbor *Arhodomonas aquaeolei* DSM 8974, an aerobic chemoheterotroph [19], and *Nitrococcus mobilis* Nb-231, an obligate chemolitotrophic bacterium [20]. The average nucleotide identity (ANI) between the two strains was only 77.3%, what fits with what is expected of different species of the same genus [21]. Both microbes have genomes close to 2 Mbp (Table 1), making them the smallest genomes described within the *Ecthiorhodospiraceae* and for any halophilic bacterium. The genomes are very streamlined with a median intergenic spacer of only 14–19 nucleotides, also the smallest of any member of this family (Figure 2). The GC content is relatively high as seems to be the case with all the *Ectothiorhodospiraceae* sequenced till now. The genomes were composed of a single circular replicon with only one rRNA operon and with a high level of synteny, considering the relatively low ANI between the two genomes (Figure 3). It is clear from the comparison with the closest available complete genome (*Alkalilimnicola ehrlichii* MLHE-1) how “*Spiribacter*” species are simplified in their metabolic versatility, missing the chemolitotrophic and carbon fixation pathways. The small number of IS and other mobile genetic elements and absence of CRISPR system and flagellum was also remarkable. All these are frequently observed characteristics of oligotrophic microbes with streamlined genomes that reach high population densities in aquatic environments, such as *Ca*. Pelagibacter ubique in the ocean.

### Ecological insights

**Figure 4:**
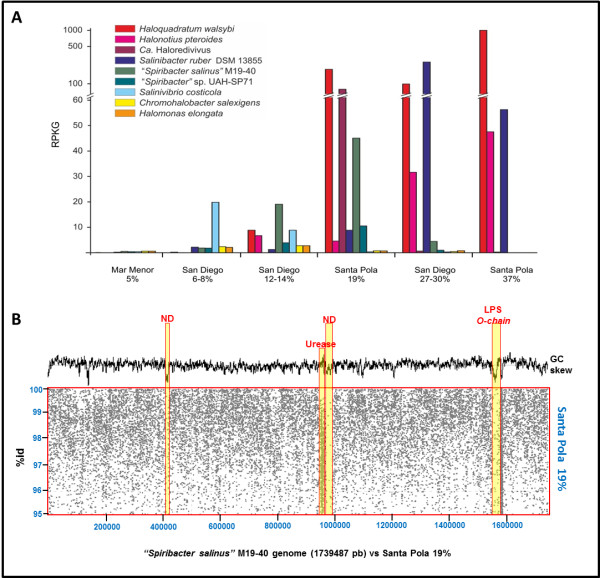
**Recruitment of some representative halophile genomes from metagenomes of hypersaline waters of different salinities. A**.- Reads recruitment at 95% of identity in at least 50 bp was normalized against the size of the genomes and the databases. Recruitment of both “*Spiribacter*” was compared with three haloarchaea: *Haloquadratum walsbyi*, *Halonotius pteroides*, *Ca*. Haloredivivus and four bacteria *Salinibacter ruber*, *Salinivibrio costicola*, *Chromohalobacter salexigens* and *Halomonas elongata*. **B**.- Figure show genomic islands and metagenomic recruitment of the “*Spiribacter salinus*” M19-40 against metagenome from Santa Pola (19%). A restrictive cut-off of 95% of nucleotide identity in 50% of the length of the metagenomic read was used. ND: not determined.

In a previous work, metagenomic sequencing indicated that the communities at 19% salinity had already a marked halophilic profile with the hyperhalophilic haloarchaeon *Haloquadratum* predominating in numbers [12]. However, a major difference with the NaCl saturated ponds was the presence of the gammaproteobacterium associated to *Alkalilimnicola*-*Nitrococcus* that we now know corresponds largely (if not completely) to “*Spiribacter*” representatives. To confirm this, we have analyzed the recruitment of the two genomes from the available metagenomes of hypersaline waters. Besides the 19% metagenome we also used data from a San Diego saltern metagenome [22] also representing intermediate salinity ponds. As shown in Figure 4, M19-40 was the known bacterial genome recruiting most in San Diego 12-14% and in Santa Pola 19%, only two halophilic archaea, *H. walsbyi* and *Ca*. Haloredivivus, recruited more. The data confirms that “*Spiribacter*” is a very abundant microbe in intermediate salinities but its abundance decreases sharply at both high and low salinities (Figure 4A). In this sense, it can be considered a *bona fide* moderate halophile that prefers to inhabit intermediate salinities. Former definitions of moderate halophiles [16] were based on laboratory studies of growth rates at different salinities, but are often misleading as shown clearly by the lack of representation of most moderate halophiles defined this way in intermediate salinities. Besides, the growth salinity range in the laboratory is often very wide, with very similar growth rates over most intermediate salinities. Some of the assembled metagenomic contigs described before were now clearly associated with “*Spiribacter*” (Additional file 1: Figure S2). Actually, these were the largest contigs obtained from the 19% metagenome confirming the abundance of “*Spiribacter*” cells in this environment. However, many of these contigs had lower similarities while still being syntenic to “*Spiribacter*” genomes (Additional file 1: Figure S2) indicating that there might be other “*Spiribacter*” species present in significant amounts in this specific sample.

The recruitment of M19-40 (Figure 4B) shows the typical metagenomic islands [23] that are found when a well-represented microbe is compared to the metagenome of its habitat. These islands are a reflection of the diversity of co-existing clonal lineages that diverge in genomic regions delineated by the islands. The most remarkable feature in the case of strain M19-40 is the small number and size of these islands concordant with the highly streamlined genome and relatively restricted metabolic versatility. Genomic island (GI) 1 accumulates 4 of the 7 IS elements of the genome but no specific function could be attributed to the few other genes present. GI2 was the largest and contains a large urease cluster. Ureases are not present in strain UAH-SP71 and, although the cluster in M19-40 recruits very little at high identity, it showed many hits below 97% identity indicating the presence of other lineages that carry ureases. The rest of GI2 contains many toxin-antitoxin systems as is typical of flexible genomic islands of bacteria in general. GI3 is the cluster coding for the O-chain of the lipopolysaccharide which is a typical genomic island in all Gram-negative bacteria and its variability has been attributed to its major role as a phage recognition target [23,24].

To assess the presence of “*Spiribacter*” associated microbes worldwide, we have searched the complete Ribosomal Database Project for 16S rRNA sequences highly similar (>97%) to that of either strain. The results described in Additional file 1: Figure S3 indicate that they are found worldwide (as already indicated by the recruitment in Spanish and Californian salterns) and always (when the data were available) in intermediate salinity hypersaline waters.

### Metabolism

Both strains of “*Spiribacter*” were isolated on media for heterotrophic aerobic microbes (M19-40 on pyruvate and yeast extract and UAH-SP71 on pyruvate, dextrose and peptone). The genomes confirm that these microbes are devoid of any photosynthetic or chemolithotrophic capabilities, what sets them apart from most members of the *Ectothiorhodospiraceae*. No carbon fixing pathways were detected either. The only other member of this family with a purely heterotrophic metabolism is *Arhodomonas aquaeolei*, a halophilic aerobic heterotroph isolated from a petroleum reservoir [19]. In concordance with their heterotrophic nature, transporters formed a large fraction of the genes in both genomes. TransporterDB (see methods) predicts 217 and 268 transport-associated genes for M19-40 and UAH-SP71, respectively. In comparison, in *S. ruber*, which has a much larger genome than either of them (3.5 Mbp), a similar number of genes (223) was detected by the same method.

One of the main carbon and energy sources for halophilic microorganisms in the salterns is glycerol. Massive amounts of this compound are produced by the unicellular green algae *Dunaliella*, the main primary producer in these hypersaline habitats, as a compatible solute to provide osmotic balance [25]. In its catabolic degradation, glycerol is converted to dihydroxyacetone phosphate (DHAP), a glycolysis intermediate, by two different pathways. The first pathway, involving glycerol kinase and glycerol 3-phosphate dehydrogenase [26], was found as a complete cluster in both “*Spiribacter*” strains. These clusters included also two genes encoding a glycerol-3-phosphate ABC transporter and a glycerol-3-phosphate regulon repressor. In strain UAH-SP71, three other different glycerol 3-phosphate dehydrogenase genes were also found together in a separate location of the genome. In the second pathway, glycerol is metabolized to DHA first and then phosphorylated by a dihydroxyacetone kinase into DHAP. In strain M19-40 this second route is totally absent while in UAH-SP71 a DHA kinase was found, indicating the ability to use both pathways. Besides, located near this DHA kinase gene, another gene coding for a phosphoenolpyruvate dependent DHA phosphotransferase system was found. Although apparently redundant, this second enzyme seems to be involved in the transport of DHA rather than in catabolism of this compound [27]. The same DHA uptake system has been found before in the haloarchaeon *H. walsbyi*[28]. The authors of that study suggested that *H. walsbyi* can use DHA as carbon and energy source, which is known to be released by *S. ruber* (and likely other halophilic microbes) as a byproduct of glycerol metabolism.

Glycine betaine (trimethylated glycine) is another important osmoprotectant that could be an abundant carbon source in hypersaline environments. Along these lines, strain M19-40 has two paralogous operons of four sarcosine oxidase genes located in separate regions of the genome. Sarcosine oxidase is a heterotetrameric enzyme that catalyzes the oxidative demethylation of sarcosine (*N*-methylglycine) to yield glycine that can be further catabolized and used as a source of carbon and energy [29]. Strain UAH-SP71 did not have these genes. Another difference indicating different lifestyles for both isolates was the presence of a complete cluster encoding a cytochrome c oxidase (cbb3) complex involved in microaerobic respiration in strain UAH-SP71. This heme-copper oxidase provides a better adaptation to respiration under microoxic conditions [30] indicating that strain UAH-SP71 might be specialized in comparatively less oxygenated microenvironments.

Regarding nitrogen metabolism, neither “*Spiribacter*” genomes have genes that code for nitrogen fixation or nitrate/nitrite assimilation. Both genomes have the gene coding for a high-affinity ammonium transporter Amt, indicating that nitrogen uptake occurs in its most reduced form, ammonia. Accordingly, the essential genes involved in ammonia assimilation and amino acid metabolism, glutamine synthetase and glutamate synthase, were found. Besides, several ABC transporters for exogenous nitrogen-rich organic compounds such as basic amino acids, putrescine/spermidine and polyamines were also found. In addition, each genome contained other non-shared genes that can provide nitrogen rich compounds. For example, strain M19-40 contained a complete urease gene cluster (see above), including the genes for the transport and conversion to ammonia, while strain UAH-SP71 carries an ABC transporter for the nitrogen rich osmolyte taurine (TauABC) [31]. Inorganic phosphate appears to be mainly transported in both microbes by the high-affinity phosphate transport system, PstSCAB. Both genomes contained also the ABC transporter PhnCDE, a high affinity uptake system for phosphonates. Phosphonates are organophosphorus compounds characterized by the presence of a carbon- phosphorus bond and could be used also as a nutritional source of phosphorus in response to phosphate starvation [32].

As expected from the evident streamlining of these genomes, regulatory mechanisms were very scarce. Only two sigma factors involved in response to environmental stresses were found, σ32 a heat shock sigma factor and σ54 that has been linked to the regulation of nitrogen metabolism [33,34]. Along similar lines, only two two-component regulatory systems were found, one involved on survival under nitrogen limited growth conditions (GlnLG) and PhoBR that regulates the response to variations in the level of extracellular inorganic phosphate [35,36].

### Osmoregulation

Despite the small genome size, both “*Spiribacter*” strains appear to have all the typical *salt*-*out* osmoregulatory mechanisms. The *salt*-*out* strategy is based in keeping most of the inorganic salts out and using organic osmolytes to balance the high salinity of the environment [37]. These “organic compatible solutes” include amino acids and derivatives such as glycine betaine and ectoine [38]. In the case of “*Spiribacter*”, judging by the number of betaine transporters in these genomes (six different ABC-type glycine betaine transport systems were found in both microbes) it seems that this compound has an important role. Besides, a choline transporter betH was also present. Choline itself is not an osmoprotectant but we found in both genomes the key enzymes for the synthesis of glycine betaine from choline, choline dehydrogenase (betA), glycine betaine aldehyde dehydrogenase (betB) and the transcriptional regulator (betI) [39]. Ectoine is another widely distributed compatible solute commonly synthesized by halophilic bacteria [40]. Ectoine biosynthesis is a separate branch along the pathway for the synthesis of the amino acid aspartate. In both “*Spiribacter*” we found the complete *ectABC* gene cluster, diaminobutyric acid (DABA) acetyltransferase (EctA), DABA aminotransferase (EctB) and ectoine synthase (EctC) [41].

**Figure 5:**
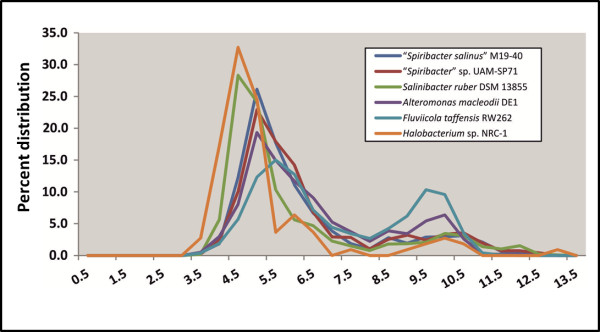
**Comparison of isoelectric profiles of “*Spiribacter*” genomes with those of other prokaryotes.** Genomes are color coded.

It has been suggested that an important adaptation to saline environments may be an increase in the number of acidic amino acids to reduce their surface hydrophobicity. We have characterized the two strains M19-40 and UAH-SP71 proteomes by virtual 2D-gels and to analyze the differences in protein acidity we have compared them to those from a salt-in halophilic archaeon (*Halobacterium* sp. NRC-1), a salt in halophilic bacterium (*Salinibacter ruber* DSM 13855), a marine bacterium (*Alteromonas macleodii* DE1) and a freshwater bacterium (*Fluviicola taffensis* RW262^T^) (Figure 5). The whole proteome pI plots showed the change from the bimodal pattern of the freshwater marine bacterium, *F. taffensis*, to a single peak around 4.5 for *Halobacterium* sp. NRC-1. This figure shows how the pI distribution of the second peak representing basic proteins, that contains the transmembrane proteins, has evolved toward acidity in response to increase in salinity of the environment. In a manner similar to *Halobacterium* sp. NRC-1, both “*Spiribacter*” showed reduction of the amount of basic amino acids resulting in a single peak at around a pI 5.0. In any case, recent evidence indicates that the low pI peak that characterizes many halophiles is more a condition for a salt-in strategy than a reflection of that mechanism really taking place in the cells [42,43].

Another way to gauge the salt adaptation of a microbe is the reliance on the Na^+^ gradient to actively transport nutrients into the cell. Both “*Spiribacter*” strains have secondary transporters that catalyze the translocation of solutes across the cytoplasmic membrane using electrochemical ion gradients, for example, the tripartite ATP-independent periplasmic family transporters [44]. Thirteen and fifteen of these transporters (in M19-40 and UAH-SP71, respectively) that catalyze uptake of C4-dicarboxylates, sugars and metabolites using the Na^+^ gradient were found. Both microbes also possess several genes encoding Na^+^ symporters for proline, bicarbonate or anions and Na^+^/H^+^ and Na^+^/Ca^2+^ antiporters, consistent with adaptations to a high-salt environment [16]. The genomes contain also a cluster of six genes coding for the multi-subunit Na^+^/H^+^ antiporter Mrp [45], which has been suggested to be the main mechanism to maintain pH homeostasis. In response to osmotic stress, bacteria can also accumulate K^+^ as an osmoregulatory solute and pH regulator [46,47]. The uptake of K^+^ is catalyzed by multiple uptake systems [48]. Interestingly, both “*Spiribacter*” strains only harbour the gene cluster *trkAH* that codes for the Trk transport system, while in *S. ruber* genome four copies of a *trkA* and two copies of a *trkH* gene and also the K^+^ efflux system KefB were present [49].

### Xanthorhodopsin (XR)

**Figure 6:**
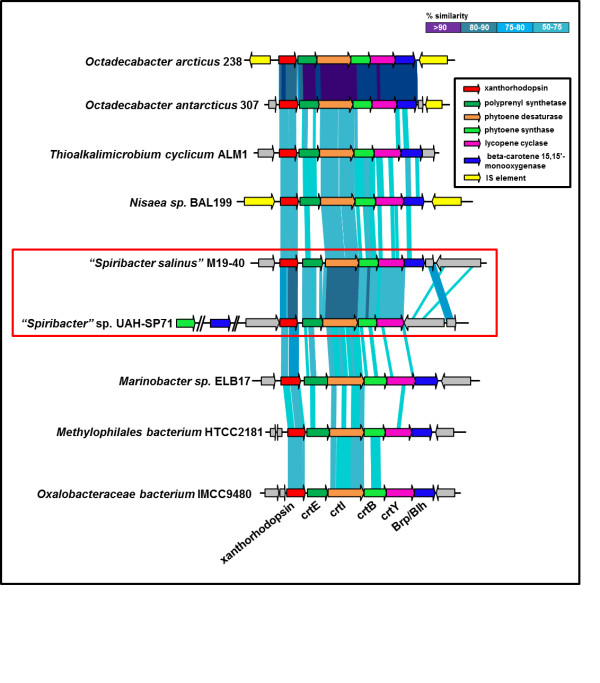
**Comparison of known xanthorhodopsin subgroup II gene cluster that includes genes involved in β-carotene and retinal synthesis.** “*Spiribacter*” clusters are highlighted in the box.

XRs are rhodopsin proton pumps that sometimes use a carotenoid pigment antenna to harvest light, a mechanism that is convergent with that of the chlorophylls of photosynthetic microbes and widens the light absorption spectrum [50]. XR was first discovered in the extremely halophilic bacterium *S. ruber*[49] but later on they have been found to be widespread in marine and halophilic microbes. Recently, a separation of xanthorhodopsins in two families has been proposed on the grounds of the gene sequence and predicted biophysical properties [51]. One of these families, Group II XRs, lack some essential keto-carotenoid binding sites and have been described as being devoid of the carotenoid antenna characteristic of group I [51]. Both “*Spiribacter*” genomes were found to contain rhodopsin-coding genes that, after sequence comparisons, cluster clearly with the XR genes found in other microbes (Additional file 1: Figure S4). The predicted proteins contained all the characteristic functional groups of proton-pumping rhodopsins (Additional file 1: Figure S5). These results suggest that both XRs are functional. Furthermore, the phylogenetic analysis (Additional file 1: Figure S4) and the characteristic genes found in the cluster (Figure 6) indicate that both closely related genes of “*Spiribacter*” encode XRs affiliated to subgroup II. This XR subgroup, was found in a heterogeneous group of different alpha and gamma proteobacteria, mostly of marine origins, and in marine eukaryotes. Actually, group II XRs have already been found by other authors in metagenomes in medium salinity, hypersaline and fresh water habitats [51].

Regardless of whether they have carotenoid antennas or not, all rhodopsins require the chromophore retinal. The gene clusters including the XR gene of group II, always contain four of the genes involved in the synthesis of the retinal precursor β-carotene from isopentenyl diphosphate (*crtE*, *crtI*, *crtB* and *crtY*) (Figure 6). In addition, UAH-SP71 has a paralogous gene (*crtB2*, 44% amino acid identity) coding for CrtB (phytoene synthase) located elsewhere in the genome. The presence of two phytoene synthase paralogs has been already reported in the genome of the Gram-positive bacterium *Corynebacterium glutamicum*[52]. The authors reported that both phytoene synthases were functional leading to an overproduction of carotenoids. Another difference between the two strains involves the location of the β-carotene 15,15'-monooxygenase (Brp/Blh) gene. In M19-40 this gene was found within the cluster while in UAH-SP71 this gene appeared at a distant locus in the genome (Figure 6).

## Conclusions

In recent years it has become apparent that planktonic aquatic habitats are often dominated by microbes that have highly streamlined genomes [2,5]. They tend to be sessile and, in the case of heterotrophs in euphotic waters, often have rhodopsins. In high-salinity hypersaline habitats, two archaeal groups appear to occupy this niche, *Haloquadratum* and the Nanohaloarchaea. In intermediate salinities there appears to be more diversity and the “*Spiribacter*” representatives fill a significant gap. The widespread distribution of this microbe and its natural range indicates that it is present in 10 to 25% salinities worldwide. In other words, it is an ecological moderate halophile in the same manner *Haloquadratum* is an ecological extreme halophile. Thus the classical classification based on growth rate range in the laboratory can be replaced by a more realistic classification based on ecological distribution. The classical microbes classified as moderate halophiles, such as *Halomonas* or *Salinivibrio*, have been shown to be almost absent from hypersaline habitats and, at least in the case of *Halomonas*, appear to be just salt tolerant opportunistic marine bacteria that happen to survive higher salinities and be thus isolated often from moderately saline waters.

“*Spiribacter*” shows many characteristics in common with dominant aquatic microbes such as *Ca*. Pelagibacter or *Ca*. Actinomarina. The cells are small, although not as small as the marine counterparts, and so is the genome. Motility is absent as in most genuinely planktonic prokaryotes. Flagellar motility only has bearing when there are significant gradients of nutrients to traverse. This is unlikely to be the case in the water column of large aquatic habitats such as the ocean. Besides, the genes involved in motility and chemotaxis impose an additional genomic burden. A major difference between the planktonic marine habitat and its hypersaline counterpart (aside from salinity itself) is the nutrient status. Hypersaline waters tend to be eutrophic and organic matter is supposed to be plentiful for heterotrophs. However, the energetic efficiency of streamlined cells such as “*Spiribacter*” has to be a significant advantage even under relatively nutrient rich conditions. *H. walsbyi* genome was not considered to be streamlined. It is a relatively large genome (*ca*. 3.0 Mbp), has very low coding density (76.5%) and contains several IS, repetitive elements [53] and genomic islands [54,55]. However, the abundance of IS elements seems to be a general feature of haloarchaea and could be related to their proven level of promiscuity [56]. Furthermore, many regions identified as non-coding have revealed a rich content of small RNAs [53] that might be very important for the biology of this microbe, i.e. the apparent wastefulness of the *Haloquadratum* genome might just reflect our ignorance of archaeal biology.

The comparison of both “*Spiribacter*” with the only other bacterium that seems to be naturally abundant in hypersaline waters, the bacteroidete *S. ruber*, is also interesting. Both bacteria are carriers of similar xanthorhodopsins, although the type present in “*Spiribacter*” seems to operate without the associated carotenoid antenna that was originally characterized in this type of rhodopsins [51]. *Salinibacter* however is motile and also carries sensory rhodopsins and chemotaxis machinery. Actually, the real relevance of *S. ruber* in the planktonic brines is not very clear. In metagenomes the available genomes do not recruit very extensively and, although its numbers are significant, they are always very far from those of the archaeal members of the population. *Salinibacter* together with many high GC haloarchaea, that are often isolated from salterns, could live attached to particles, a niche that is often overlooked but can explain the need for motility to commute between particles or get to the most nutrient rich areas.

## Methods

### Isolation of the strains

The two strains used in this study were isolated from water samples collected from two intermediate salinity ponds of the salterns “Braç del Port” located in Santa Pola (Alicante, Spain, 38º13´ N - 0º35´ W) and Isla Cristina (Huelva, Spain, 37°13´ N – 7°19´ W). “*Spiribacter*” sp. UAH-SP71 isolated from the Santa Pola salterns was grown in medium R2 Agar (Microkit, ref. DMT215) contained (in %): 0.0525 peptone, 0.035 yeast extract, 0.035 dextrose, 0.035 starch, 0.021 K_2_HPO_4_, 0.021 sodium pyruvate, 0.0175 tryptone, 0.00168 MgSO_4_ and supplemented with 10% of sea salts [57], pH 8. The medium for “*Spiribacter salinus*” M19-40 contained in % 15 sea salts, 0.5 casein digest, 0.1 yeast extract, 0.1 dextrose and 0.1 pyruvic acid, pH 7.5.

### DNA sequencing and assembly

DNA was extracted by phenol-chloroform [58] and checked for quality on a 1% agarose gel. Whole genome sequencing for both isolates was performed using the Illumina HiSeq 2000 (100-bp paired-end read) sequencing platform. In addition, Pacific Bioscience 3- to 5-Kb reads were generated for “*Spiribacter salinus*” M19-40. Error correction of the PacBio reads was performed using the Illumina reads as described before [59]. The reads were assembled de novo using MIRA [60] and Geneious Pro 5.0.1. Both results were compared for equal assemblies. Finally, oligonucleotides designed from the sequence of the ends of assembled contigs were used to obtain single closed contigs [61].

### Genome annotation

The genomes were annotated using the NCBI PGAAP annotation pipeline (http://www.ncbi.nlm.nih.gov/genome/annotation_prok/). The predicted protein sequences were also compared using BLASTP to the National Center for Biotechnology Information nr protein database (e value: 1e-5). BioEdit was used to manipulate the sequences [62]. GC content was calculated using the EMBOSS tool geecee [63]. For comparative analyses, reciprocal BLASTN and TBLASTXs searches between the genomes were carried out, leading to the identification of regions of identity, insertions and rearrangements. To allow the interactive visualization of genomic fragment comparisons Artemis v.12 [64], Artemis Comparison Tool ACTv.9 [65] were used to compare the genomes. Average nucleotide identity (ANI) was calculated as defined before [21]. Transporters were annotated using the TransporterDB database [66].

### Phylogenetic analysis

To determine the exact phylogenetic relationship of the new isolates within the family, phylogenetic analysis of 16S rRNA gene sequences for all the *Ectothiorhodospiraceae* members were carried out. Maximum likelihood tree was created using MEGA (version 4.0.2). The tree was rooted using *Allochromatium vinosum* DSM 180 and *Thioflavicoccus mobilis* 8321 as outgroups. For creating the phylogenomic tree in Figure 1, the complete genomes of *Ectothiorhodospiraceae* were analyzed using TIGRfams and 277 proteins conserved in all genomes were identified and concatenated. The concatenated proteins were aligned using Kalign [67] and a maximum likelihood tree was made using FastTree [68] using a JTT + CAT model and a gamma approximation.

### Fragment recruitment

Genomes recruitments from metagenomic datasets derived from some available marine habitats with different salinity [12,22] were carried out via BLASTN. A restrictive cut-off of 95% of identity in at least 50 bp was established to guarantee that only similarities at the level of nearly identical microbes were counted. The number of hits was normalized against the genomes and the database sizes. As controls, similar recruitment experiments were carried out for other typical halophilic microorganism in this environment, three haloarchaea: *Haloquadratum walsbyi*, *Halonotius pteroides*, *Ca*. Haloredivivus and four bacteria *Salinibacter ruber*, *Salinivibrio costicola*, *Chromohalobacter salexigens* and *Halomonas elongata*. Recruitment of the genome of strain M19-40 against the metagenome from Santa Pola (19%) (Figure 4B), 95% of identity in 50% of the length of the metagenomic read was used as a cut-off to construct the plot.

### Accessions

The genomes have been deposited in NCBI GenBank and can be accessed with the following accession numbers: “*Spiribacter salinus*” M19-40 (CP005963) and “*Spiribacter*” sp. UAH-SP71 (CP005990).

## Competing interests

The authors declare that they have no competing interests.

## Authors’ contributions

FRV, AV and JS conceived the study, and participated in its design and coordination. ML, ARO, JLCP and CSP isolated the strains. Assembly and genome analysis was carried out by MLP, RG and MJL. FRV, MLP and RG wrote the manuscript. All authors read and approved the final manuscript.

## Supplementary Material

Additional 1 **Figure S1.** 16S rRNA phylogeny. Maximum likelihood phylogenetic tree based on the comparison of 16S rRNA gene sequences of the *Ectothiorhodospiraceae. Allochromatium vinosum* DSM 180 and *Thioflavicoccus mobilis* 8320, belonging to the *Chromatiaceae* were used as outgroup and are shown in red. Bootstrap values are indicated at the nodes. **Figure S2.** BLASTN comparisons of metagenomic contigs from 19% Santa Pola to “*Spiribacter*” genomes. The metagenomic contigs are shown in the middle. A color key for the similarity is shown on the top right. **Figure S3.** Global distribution of Spiribacter 16S rRNA gene sequences. Locations where 16S rRNA gene sequences from the Ribosomal Database Project were found (>97% identical, >300 bp) are indicated by colored boxes. The color code indicates the number of sequences found at each location. The map is a modified version of a freely available map from http://www.naturalearthdata.com. **Figure S4.** Phylogenetic tree of the two xanthorhodopsins found in both “*Spiribacter*” with all the xanthorhodopsins available. Taxonomy and origin of isolation of each strain are also shown. **Figure S5.** Xanthorhodopsin amino acid sequence alignment. Multiple alignments of all the predicted aminoacid sequences of the two xanthorhodopsin subgroups. Rectangles over the sequence indicate predicted transmembrane regions. Proton acceptor and donor and the conserved lysine linked to the cofactor retinal are marked by a rectangle with a yellow line. Yellow rectangles with red line indicated the residues that interact with the keto-carotenoid identified by [69]. Maintaining nomenclature, the letters c, g, k and r, indicate contact with the chain, glucoside, keto group and ring of the carotenoid, respectively. Click here for file
