# Supplementary material for: Genomes of “Spiribacter”, a streamlined, successful halophilic bacterium
Source: BMC Genomics. 2013 Nov 13;14:787. doi: 10.1186/1471-2164-14-787 (PMC3832224; doi:10.1186/1471-2164-14-787)
Supplement: Additional 1 — Figure S1. 16S rRNA phylogeny. Maximum likelihood phylogenetic tree based on the comparison of 16S rRNA gene sequences of the Ectothiorhodospiraceae. Allochromatium vinosum DSM 180 and Thioflavicoccus mobilis 8320, belonging to the Chromatiaceae were used as outgroup and are shown in red. Bootstrap values are indicated at the nodes. Figure S2. BLASTN comparisons of metagenomic contigs from 19% Santa Pola to “Spiribacter” genomes. The metagenomic contigs are shown in the middle. A color key for the similarity is shown on the top right. Figure S3. Global distribution of Spiribacter 16S rRNA gene sequences. Locations where 16S rRNA gene sequences from the Ribosomal Database Project were found (>97% identical, >300 bp) are indicated by colored boxes. The color code indicates the number of sequences found at each location. The map is a modified version of a freely available map from http://www.naturalearthdata.com. Figure S4. Phylogenetic tree of the two xanthorhodopsins found in both “Spiribacter” with all the xanthorhodopsins available. Taxonomy and origin of isolation of each strain are also shown. Figure S5. Xanthorhodopsin amino acid sequence alignment. Multiple alignments of all the predicted aminoacid sequences of the two xanthorhodopsin subgroups. Rectangles over the sequence indicate predicted transmembrane regions. Proton acceptor and donor and the conserved lysine linked to the cofactor retinal are marked by a rectangle with a yellow line. Yellow rectangles with red line indicated the residues that interact with the keto-carotenoid identified by [69]. Maintaining nomenclature, the letters c, g, k and r, indicate contact with the chain, glucoside, keto group and ring of the carotenoid, respectively. [file 1471-2164-14-787-S1.pdf]

Genomes of "*Spiribacter*", a streamlined, successful halophilic bacterium

Mario López-Pérez[1], Rohit Ghai[1], Maria Jose Leon[2], Ángel Rodríguez-Olmos[3], José Luis Copa-Patiño[3], Juan Soliveri[3], Cristina Sanchez-Porro[2], Antonio Ventosa[2], and Francisco Rodriguez-Valera[1*]

[1]Evolutionary Genomics Group, División de Microbiología, Universidad Miguel Hernández, Apartado 18, San Juan 03550, Alicante, Spain

[2]Department of Microbiology and Parasitology, Faculty of Pharmacy, University of Sevilla, Sevilla, Spain

[3]Department of Microbiology and Parasitology, Faculty of Pharmacy, University of Alcalá, 28871 Alcalá de Henares, Madrid, Spain

*Address correspondence to Francisco Rodriguez-Valera, frvalera@umh.es

Evolutionary Genomics Group, División de Microbiología, Universidad Miguel Hernández, Apartado 18, San Juan 03550, Alicante, Spain

Phone +34-965919313, Fax +34-965 919457

Running Head: "*Spiribacter*", a streamlined halophile

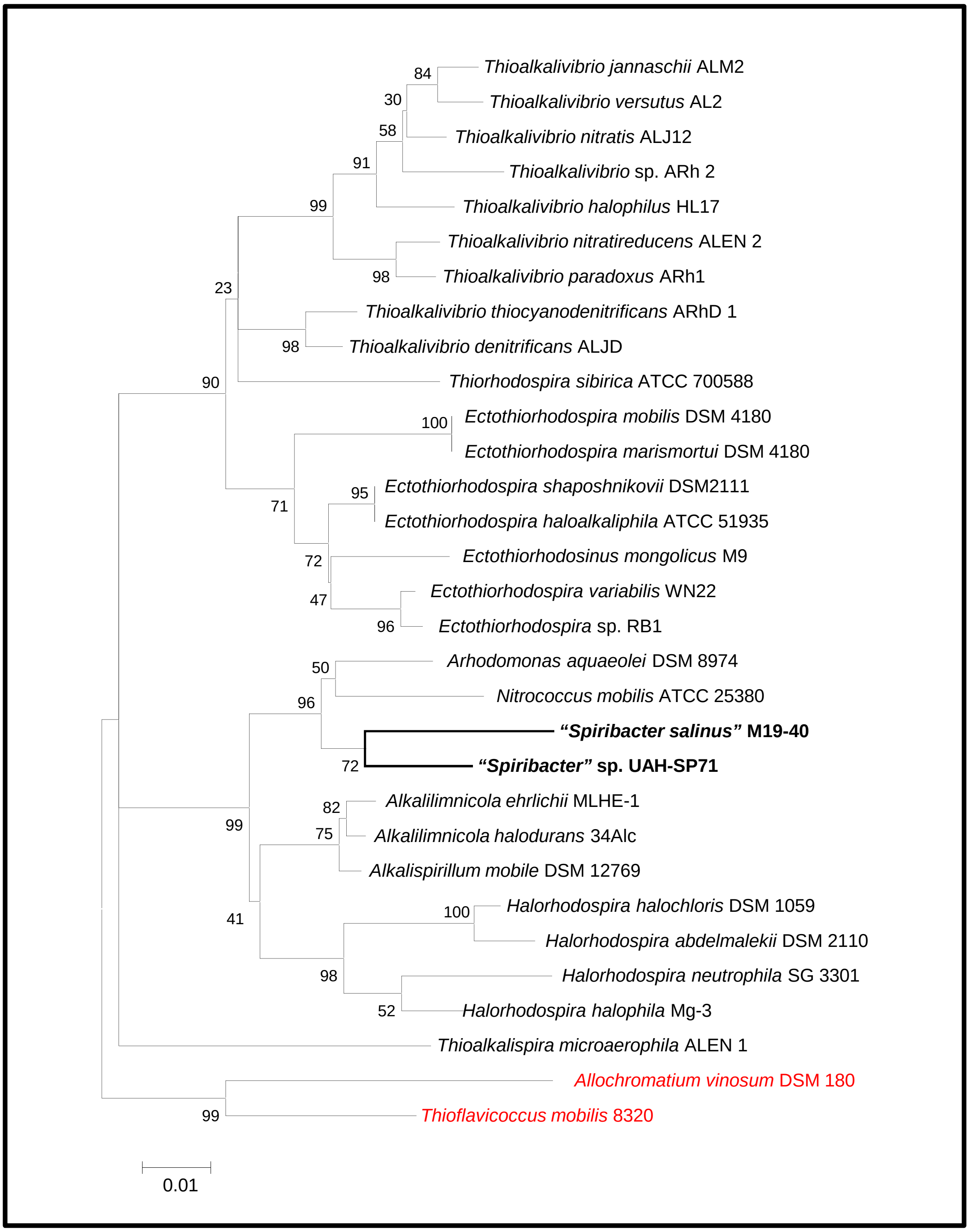

Additional Figure S1: 16S rRNA phylogeny. Maximum likelihood phylogenetic tree based on the comparison of 16S rRNA gene sequences of the *Ectothiorhodospiraceae. Allochromatium vinosum* DSM 180 and *Thioflavicoccus mobilis* 8320, belonging to the *Chromatiaceae* were used as outgroup and are shown in red. Bootstrap values are indicated at the nodes.

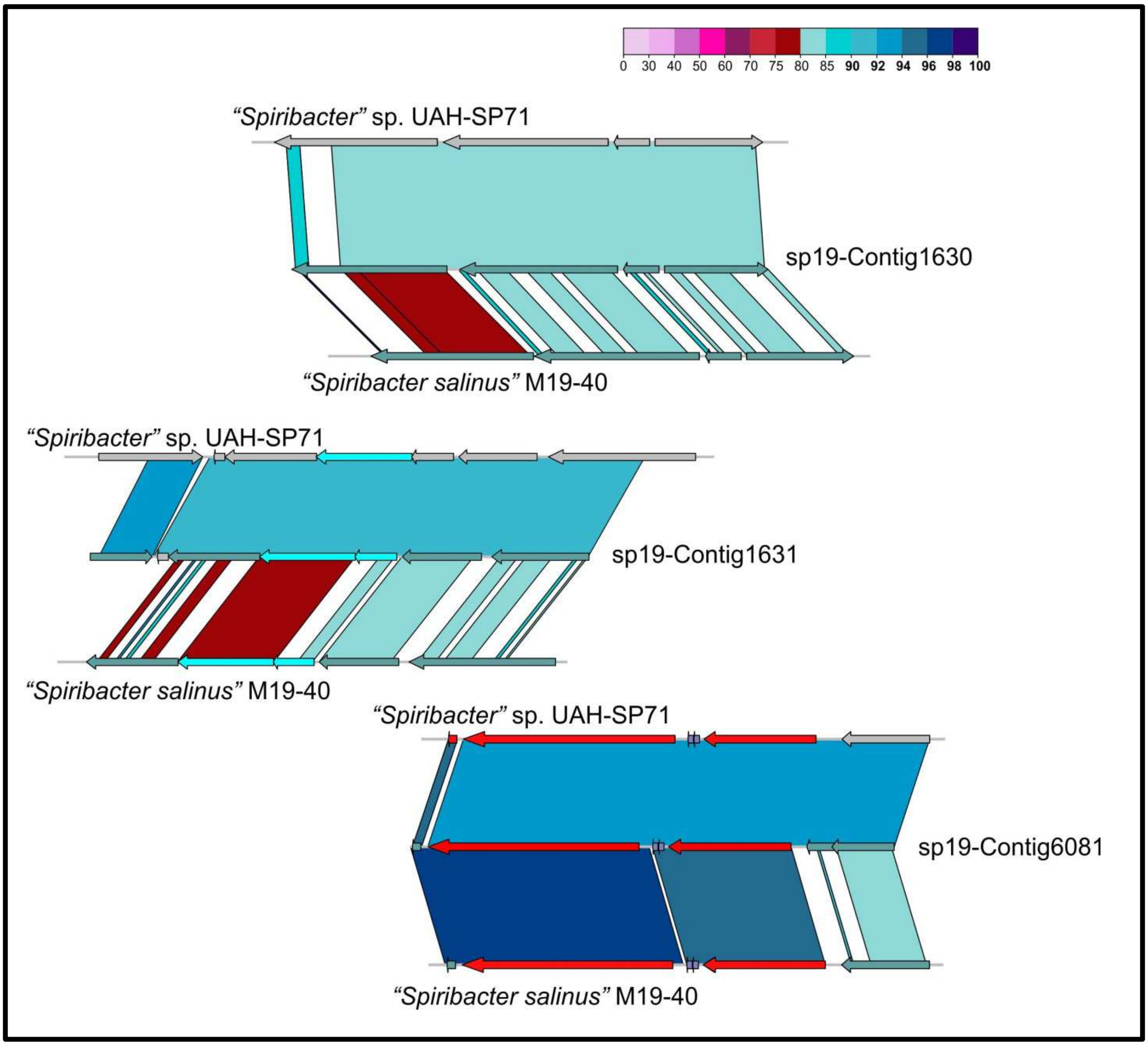

Additional Figure S2: BLASTN comparisons of metagenomic contigs from 19% Santa Pola to "*Spiribacter*" genomes. The metagenomic contigs are shown in the middle. A color key for the similarity is shown on the top right.

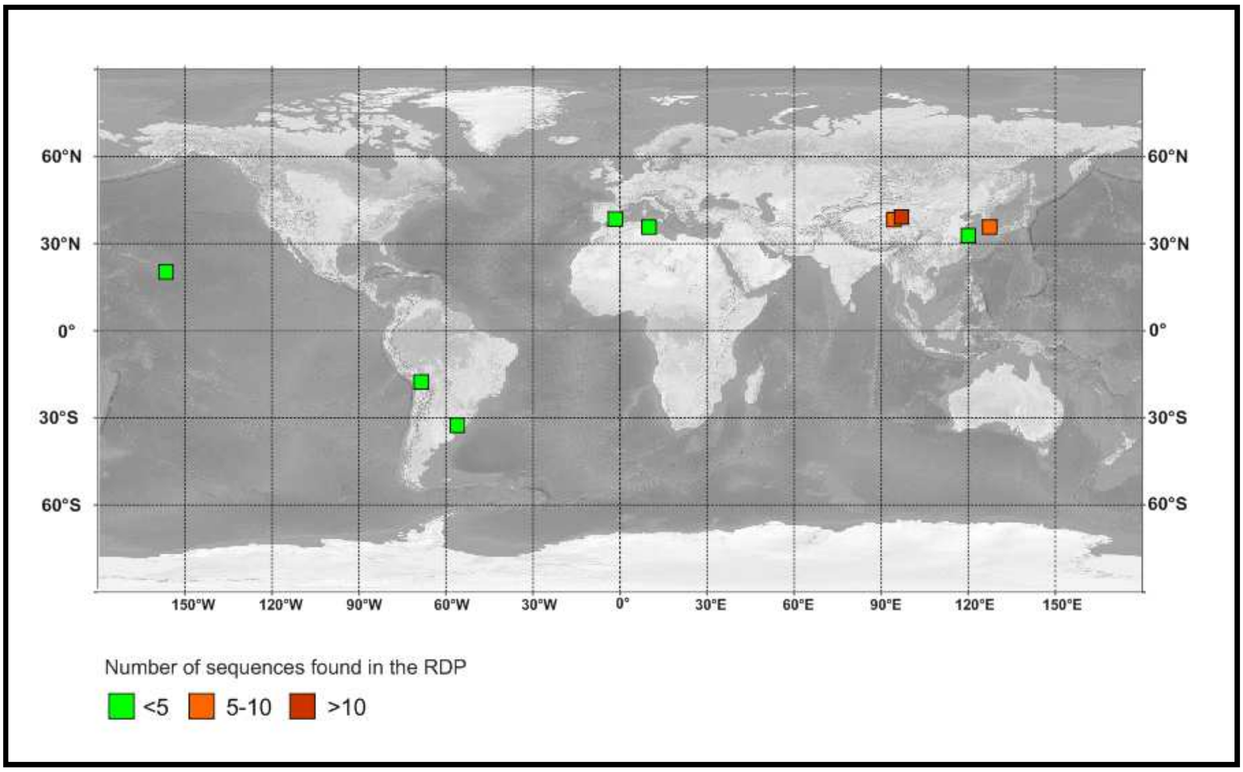

Additional Figure S3: Global distribution of Spiribacter 16S rRNA gene sequences. Locations where 16S rRNA gene sequences from the Ribosomal Database Project were found (>97% identical, >300 bp) are indicated by colored boxes. The color code indicates the number of sequences found at each location. The map is a modified version of a freely available map from http://www.naturalearthdata.com.

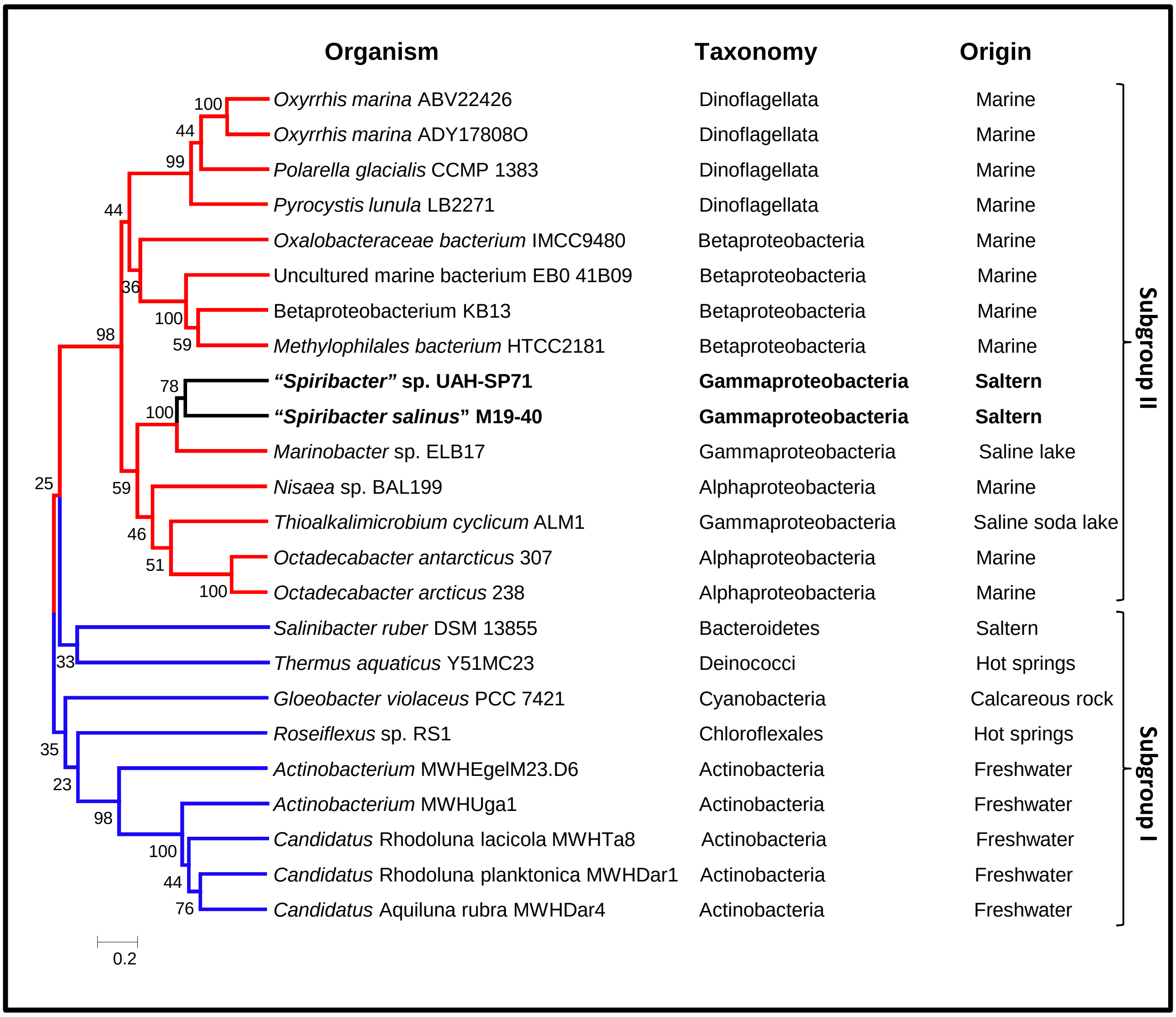

Additional Figure S4: Phylogenetic tree of the two xanthorhodopsins found in both "*Spiribacter*" with all the xanthorhodopsins available. Taxonomy and origin of isolation of each strain are also shown.

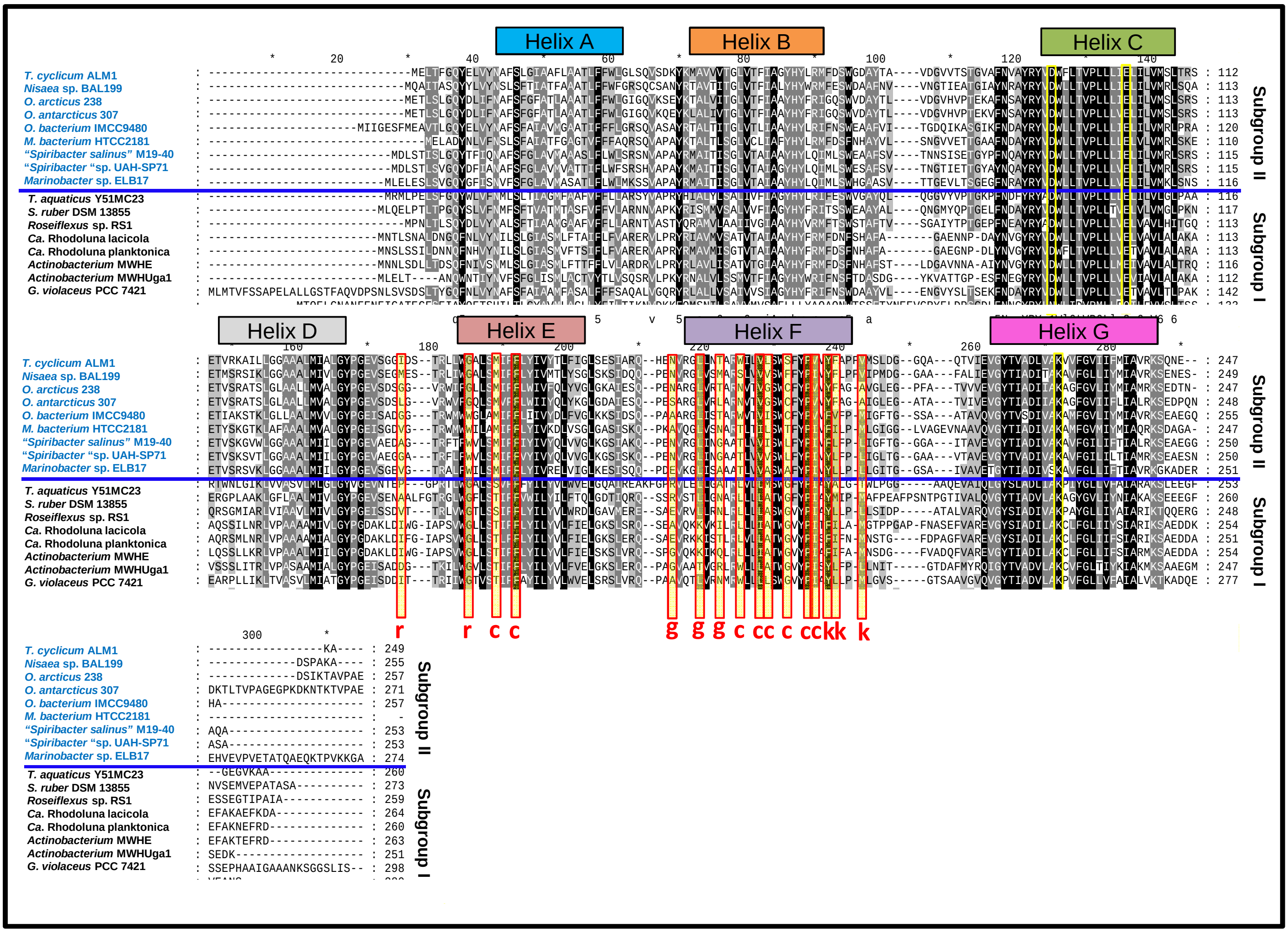

Additional Figure S5: Xanthorhodopsin amino acid sequence alignment. Multiple alignments of all the predicted aminoacid sequences of the two xanthorhodopsin subgroups. Rectangles over the sequence indicate predicted transmembrane regions. Proton acceptor and donor and the conserved lysine linked to the cofactor retinal are marked by a rectangle with a yellow line. Yellow rectangles with red line indicated the residues that interact with the keto-carotenoid identified by [69]. Maintaining nomenclature, the letters c, g, k and r, indicate contact with the chain, glucoside, keto group and ring of the carotenoid, respectively.
